# Combined proteomic and metabolomic analyses of cerebrospinal fluid from mice with ischemic stroke reveals the effects of a Buyang Huanwu decoction in neurodegenerative disease

**DOI:** 10.1371/journal.pone.0209184

**Published:** 2019-01-15

**Authors:** Wei-Hsiang Hsu, Yuh-Chiang Shen, Young-Ji Shiao, Ching-Hua Kuo, Chung-Kuang Lu, Tai-Yuan Lin, Wei-Chi Ku, Yun-Lian Lin

**Affiliations:** 1 Department of Chinese Pharmaceutical Sciences and Chinese Medicine Resources, China Medical University, Taichung, Taiwan; 2 National Research Institute of Chinese Medicine, Ministry of Health and Welfare, Taipei, Taiwan; 3 Department of Pharmacy, National Taiwan University, Taipei, Taiwan; 4 School of Medicine, College of Medicine, Fu Jen Catholic University, New Taipei, Taiwan; University of Florida, UNITED STATES

## Abstract

Ischemic stroke is one of the most common causes of death worldwide and is a major cause of acquired disability in adults. However, there is still a need for an effective drug for its treatment. Buyang Huanwu decoction (BHD), a traditional Chinese medicine (TCM) prescription, has long been used clinically to aid neurological recovery after stroke. To establish potential clinical indicators of BHD efficacy in stroke treatment and prognosis, we conducted a combined proteomic and metabolomic analysis of cerebrospinal fluid (CSF) samples in a mouse stroke model. CSF samples were obtained from male mice with acute ischemic stroke induced by middle cerebral ischemic/reperfusion (CI/R) injury, some of which were then treated with BHD. Label-free quantitative proteomics was conducted using nano-LC-MS/MS on an LTQ Orbitrap mass and metabolomic analysis was performed using nanoprobe NMR and UHPLC-QTOF-MS. The results showed that several proteins and metabolites were present at significantly different concentrations in the CSF samples from mice with CI/R alone and those treated with BHD. These belonged to pathways related to energy demand, inflammatory signaling, cytoskeletal regulation, Wnt signaling, and neuroprotection against neurodegenerative diseases. In conclusion, our *in silico* data suggest that BHD treatment is not only protective but can also ameliorate defects in pathways affected by neurological disorders. These data shed light on the mechanism whereby BHD may be effective in the treatment and prevention of stroke-related neurodegenerative disease.

## Introduction

Cerebral stroke, a sudden interruption in the blood supply to the brain, is a major cause of death and long-term disability globally. Most strokes are caused by an abrupt blockage of arteries, resulting in cerebral ischemia [1]. Such ischemic strokes can impair sensory processing, communication, cognition, and motor function in patients, as well as imposing a heavy social burden [2]. In addition, stroke increases the risk of neurodegenerative diseases, like vascular dementia (VaD), Parkinson’s and Alzheimer’s diseases (AD) [3, 4]. At present, tissue plasminogen activator (TPA) is the only effective drug for use in a limited group of patients in the acute phase of ischemic stroke [5]. Thus, there is an unmet need for additional safe and effective treatments that could improve the outcomes following stroke, which might be corrected by surveys of candidates used in complementary and/or alternative medicine.

Buyang Huanwu decoction (BHD), a well-known traditional Chinese medicine (TCM) prescription, has long been utilized to improve the recovery of neurological function in patients with paralysis and stroke [6]. Recent pharmacological studies have revealed some of the molecular mechanisms of the neuroprotective effect of BHD, including an anti-inflammatory action, the improvement of cerebral microcirculation, and a reduction in free-radical concentrations [7, 8]. Our previous studies in mice with cerebral ischemic/reperfusion (CI/R) injury showed that BHD treatment restored the concentrations of most abnormal metabolites, preserved the integrity of the blood–brain barrier (BBB), suppressed cytotoxicity, and enhanced energy metabolism, suggesting multiple effects of BHD in this model [9, 10].

Given the complexity and heterogeneity of stroke etiology, it has been suggested that a multiplexed panel of biomarkers for body fluids with specific and complementary characteristics may assist in the diagnosis, risk assessment, treatment selection, and prediction of clinical outcomes in patients with brain damage. To date, few biomarkers or molecular therapeutic targets for ischemic stroke have been used in clinical practice [11]. Cerebrospinal fluid (CSF), in which ~20% proteins are derived from the brain, holds great potential for identifying brain-specific biomarkers [12]. Because BHD has been widely used as an alternative and effective remedy for stroke sequelae in TCM [6], we hypothesized that a comprehensive study of the molecular characteristics of CSF could provide clinical indicators of the response to stroke treatment with BHD and the associated prognosis. Therefore, integrative proteomic and metabolomic analyses of CSF samples from CI/R-induced–stroke mice were performed to explore the potential neuroprotective mechanisms associated with BHD treatment. Our results provide molecular evidence for the beneficial effects of BHD in ischemic stroke.

## Materials and methods

### Chemicals and herbal materials

All chemicals, unless otherwise stated, were purchased from Sigma (St. Louis, MO). MS-grade trypsin protease was obtained from Thermo Fisher Scientific (Rockford, IL). Deionized water (18.1 μΩ cm resistivity) from a Milli-Q system (Millipore, Bedford, MA) was used throughout this study.

Buyang Huanwu decoction (BHD) was prepared from Hongqi (Hedysari Radix), Dangguiwei (Angelicae Sinensis Radix), Chishao (Paeoniae Rubra Radix), Chuanxiong (Chuanxiong Rhizoma), Taoren (Persicae Semen), Honghua (Carthami Flos), and Dilong (Pheretima), which were mixed in order at a ratio of 120:10:10:10:10:10:4.5. Herb identification, drug preparation, and HPLC fingerprinting were performed as previously described, *i*.*e*. the same batch of BHD was used as our previous studies [9, 10]. The chemical fingerprint of BHD is shown in S1 Fig. The lyophilized BHD was resuspended in normal saline to a final concentration of 2.0 g/mL (equivalent to the dry weight of the raw materials) for animal administration.

### Statement of animal ethics

All animal treatment procedures were performed under The Guide for the Care and Use of Laboratory Animals (NIH publication, 85–23, revised 1996) as well as the ARRIVE guidelines, and were reviewed and approved by the Animal Research Committee of the National Research Institute of Chinese Medicine, Taipei, Taiwan, under IACUC protocol no. P-99-11 and IACUC Approval No: A-99-1, respectively. All surgeries were performed under anesthesia with all efforts to minimize animal suffering. CSF samples were collected at day after the induction of stroke, and the mice were euthanized as pre-specified the humane endpoints.

### Animal groupings and treatments

Six-week-old male ICR mice, purchased from the National Laboratory Animal Breeding and Research Center, Taipei, Taiwan, were housed individually and fed a laboratory standard diet (Lab Rodent Chow Die 5001, Ralston Purina Co. St. Louis, Mo) *ad libitum* for one week. Twenty-seven mice were randomly divided into three groups: (I) sham control (n = 9), (II) middle cerebral ischemic/reperfusion (CI/R) injury (n = 9), and (III) CI/R followed by BHD treatment (CI/R+BHD) (n = 9).

CI/R injury were induced as previously described [9, 10], and based on the Stroke Therapy Academic Industry Roundtable (STAIR) recommendations [13]. According to our prior dose–response study, 1.0-g/kg oral BHD was determined to be the optimal dose [14]; therefore, we used this in the current study. Briefly, 2 h after the induction of CI/R, mice were administered with either 1.0 g/kg BHD (CI/R+BHD group) or vehicle (CI/R and sham groups) orally. Subsequent doses of BHD were administered every 12 h. During the treatment period the mice had *ad libitum* access to food and water.

### CSF collection and preparation

CSF collection was performed according to the method previously described [15] on the day following stroke. Briefly, mice were anesthetized by intraperitoneal injection of ketamine (100 mg/kg) and xylazine (10 mg/kg), and maintained in a 37°C incubator during induction. The anesthetized mice were placed horizontally with their cisternae magnae surgically exposed. CSF samples were collected using a capillary tube with a tapered tip that was inserted through the exposed meninges. Fifty to sixty microliters of CSF were collected from each mouse and stored at −80°C for analysis. Two independent batches of CSF samples were collected following the animal treatments.

### Evaluation of infarct volume after CI/R injury

Immediately after CSF collection, the mice were sacrificed under deep anesthesia by intraperitoneal injection of a mixture of zolazepam (50 mg/kg) and xylazine (10 mg/kg). Their entire brains were rapidly removed and 1-mm-thick coronal sections were prepared for staining with 2,3,5-triphenyltetrazoliumchloride (Sigma-Aldrich, St. Louis, MO, USA). The brain slices were then photographed to determine infarct volumes, which were corrected for the degree of edema, as described previously [10].

### Proteomic analyses of CSF samples

CSF samples from three mice per treatment group were pooled and the seven most abundant proteins in the CSF were immunodepleted using Seppro Mouse Spin Columns (Sigma-Aldrich). The immunodepleted CSF samples were then digested and fractionated, as previously described [16]. The depleted proteins were resuspended in a digestion buffer, heated at 95°C for 5 min, and then digested using trypsin at a protein/trypsin ratio (w/w) of 50:1 at 37°C overnight. The digested peptides were then desalted using an SDB-RPS membrane (3M, St. Paul, MN, USA), and eluted into three fractions.

Nano-LC-MS/MS was performed using an LTQ Orbitrap XL mass spectrometer (Thermo Fisher Scientific, Bremen, Germany). The dried fractionated peptides were resuspended in 0.1% formic acid, and loaded onto an in-house-prepared 100 μm × 15 cm column packed with 3-μm ReproSil-Pur 120 C18-AQ reverse-phase beads (Dr. Maisch HPLC GmbH, Ammerbuch-Entringen, Germany). The detailed LC conditions and instrument parameters are described in S1 Table. All the raw proteomic data obtained have been deposited with the ProteomeXchange Consortium via the PRIDE partner repository, with the dataset identifier PXD006342.

### Metabolomic profiling of CSF samples

^1^H-NMR analysis was performed largely as described by Chen *et al* [10]. Briefly, 30 μl of CSF in 10 μl of cold D_2_O containing 0.9% sodium chloride and 0.375% TSP-*d*_*4*_ was placed in an NMR nanotube for analysis. The T2 relaxation-edited CPMG sequence (90-(τ-180-τ)_n_-acquire) was used to record ^1^H-NMR spectra. These spectra were phase and baseline-corrected and then referenced to the internal TSP at δ 0.00 ppm using Topspin (Bruker Topspin 2.1).

The metabolomic UHPLC-QTOF-MS profiling experiments were conducted using an Agilent 1290-UHPLC coupled with an Agilent 6540 QTOF mass system (Agilent, Santa Clara, CA, USA). Further details of this refer to a specific Supporting Information file (i.e. S1 Table). MS raw files were converted into the mzXML format using Trapper (Institute for Systems Biology [ISB]). The mzXML data were then processed using an in-house package, TIPick, which has been developed to objectively remove background signals, and to detect and enhance user-specified metabolites from UHPLC-MS data. Statistical analysis and interpretation were focused only on the TIPick-identified metabolites. After TIPick processing, scaling-based normalization was performed according to the total ion abundances in the UHPLC-MS data.

### Statistical analysis of CSF metabolomic data

SIMCA-P+ (version 12.0, Umetrics, Umeå, Sweden) was used to process the ^1^H-NMR spectrum datasets. For multivariate statistical analysis, PCA, PLS-DA, and OPLS-DA were employed to analyze the covariance between the measured peak intensities in the MS or NMR spectra and the response variable.

### Bioinformatics analyses

Biological pathway and functional annotation of the proteomic and metabolomic data were performed using PANTHER [17], Ingenuity Pathway Analysis (IPA) software (Ingenuity Systems, Mountain View, CA), and Metabolomics Pathway Analysis (MetPA) [18].

### Western blot

Twenty micrograms of CSF proteins were separated using sodium dodecyl sulfate-polyacrylamide gel electrophoresis and then transferred to polyvinylidene difluoride membranes using a Bio-Rad transfer system. Following the transfer, the membranes were stained with Ponceau S to confirm the efficiency and uniformity of the protein transfer. The membranes were blocked with 5% nonfat skim milk at room temperature for 30 min and then incubated with a primary antibody at 4°C overnight. After this, the membranes were incubated with horseradish-peroxidase (HRP)–conjugated secondary antibodies at a dilution of 1:8,000 for 2 h at room temperature. The immunoreactive protein bands were then visualized using HRP substrate peroxide solution/luminol reagents (Immobilon Western Chemiluminescent Substrate, Millipore; mixed in a 1:1 ratio) and recorded with the Fujifilm LAS4000 luminescent image analysis system. The following primary antibodies were used: anti-amyloid beta precursor like protein (APLP1), anti-stathmin 1 (STMN1), anti-dickkopf-related protein 3 (DKK3), anti-carboxylesterase 1C (CES1C), anti-serpin family B member 5 (SERPINB5), and anti-alpha-2-macroglobulin (A2M) (GeneTex); anti-secretogranin 3 (SCG3), anti-brevican (BCAN), and anti-neuronal pentraxin receptor (NPTXR) (Santa Cruz Biotechnology); anti-cell adhesion molecule 4 (CADM4) and anti-epiplakin 1 (EPDR1) (Abcam); and anti-heat shock protein family A (Hsp70) member 1A (HSPA1A), anti-junction plakoglobin (JUP), and anti-S100 calcium binding protein B (S100B) (Abnova). All primary antibodies were used at a dilution of 1:1,000.

## Results

### Proteomic profiling of CSF by label-free quantitative proteomics

We first measured CSF protein concentrations in CI/R mice that were or were not treated with BHD. Because low concentrations of brain-derived proteins in the CSF (at the ng/mL level) could be masked by highly abundant proteins, immunodepletion was performed to remove the seven most abundant proteins. Label-free quantitative proteomic analysis [19], identified a total of 5,523 unique peptides, corresponding to 818 proteins, and 344 of these were successfully quantified (S1 Table). Of these, 144 candidate proteins in 132 protein families demonstrated significant differences (false discovery rate < 0.05) in at least one group (sham, CI/R, or CI/R+BHD mice) (S1 Table).

**Fig 1:**
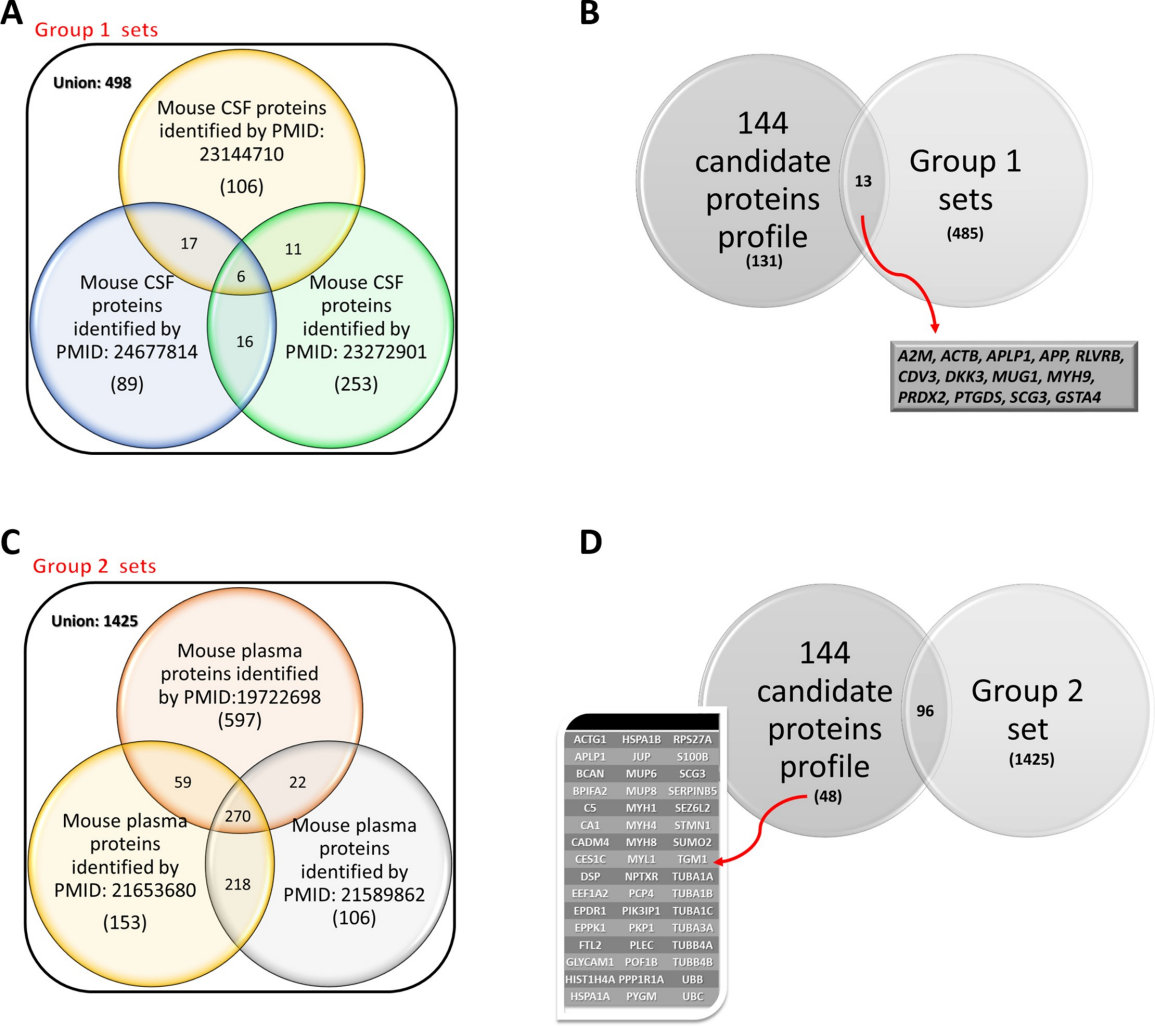
Venn diagram with CSF of CI/R-induced stroke compartmentalization. (A-B) Comparison of unique proteins identified in murine CSF with previously reported unique proteins in the CSF [20–22]. (C-D) Comparison of unique proteins identified in murine CSF with previously reported unique proteins in the plasma [23–25].

To obtain the most representative protein candidates from the CSF, we used a two-step filtering process. First, a dataset containing 498 CSF proteins (Group 1) extracted from three studies of mouse CSF [20–22] (Fig 1A) were compared with the 144 protein candidates. Thirteen proteins that were present in both datasets (Fig 1B) were considered to be “known” CSF proteins. Second, we tried to decrease the number of proteins related to blood contamination, which are usually included during the CSF sampling process, by filtering the 144 protein candidates using another dataset containing 1,425 mouse plasma proteins (Group 2) identified in three previous studies [23–25] (Fig 1C). After removing the potentially plasma-derived proteins, the remaining 48 protein candidates (Fig 1D) were selected and regarded as CSF-specific proteins. Finally, we combined the 13 and 48 filtered proteins into a new list containing 59 CSF proteins (Table 1), which we regarded as CI/R-changed CSF proteins that could be potentially involved in the protective effect of BHD in CI/R-induced cerebral stroke.

**Table 1:** Characterization of selected protein candidates in CIR–induced, sham and BHD treatment ischemic stroke mice.

Protein IDs (UniProtKB)	Gene Symnol	Description	Related pathway (PANTHER)	CIR/Sham, Log_2_		(CIR+BHD)/Sham, Log_2_	
**Q61838**	A2M	Alpha-2-macroglobulin;Alpha-2-macroglobulin 165 kDa subunit	1. Blood coagulation 2. Inflammation mediated by chemokine and cytokine signaling pathway 3. Interleukin signaling pathway	-1.35	**	0.11	^##^
**P60710**	ACTB	Actin, cytoplasmic 2; Actin, cytoplasmic 2, N-terminally processed	1. Alzheimer disease-presenilin pathway 2. Cadherin signaling pathway 3. Cytoskeletal regulation by Rho GTPase 4. Huntington disease 5. Inflammation mediated by chemokine and cytokine signaling pathway 6. Integrin signalling pathway 7. Nicotinic acetylcholine receptor signaling pathway 8. Wnt signaling pathway	-0.38	**	1.19	^##^
**P63260**	ACTG1	Actin, cytoplasmic 1; Actin, cytoplasmic 1, N-terminally processed	1. Alzheimer disease-presenilin pathway 2. Cadherin signaling pathway 3. Cytoskeletal regulation by Rho GTPase 4. Huntington disease 5. Inflammation mediated by chemokine and cytokine signaling pathway 6. Integrin signalling pathway 7. Nicotinic acetylcholine receptor signaling pathway 8. Wnt signaling pathway	-0.38	**	1.19	^##^
**Q03157**	APLP1	Amyloid-like protein 1	Alzheimer disease-presenilin pathway	0.11		-0.70	^#^
**P12023**	APP	Amyloid precursor protein	Alzheimer disease-presenilin pathway	-0.09		-1.12	^#^
**Q61361**	BCAN	Brevican core protein	Alzheimer disease-presenilin pathway	0.39		-0.92	^##^
**Q923D2**	BLVRB	Flavin reductase (NADPH)	Interleukin signaling pathway ATP synthesis	0.35		-0.86	^##^
**P07743**	BPIFA2	BPI fold-containing family A member 2	——	1.00	**	1.25	
**P06684**	C5	Complement C5	1. Inflammation mediated by chemokine and cytokine signaling pathway	-0.77	**	0.41	^##^
**P13634**	CA1	Carbonic anhydrase 1	——	0.37	**	-0.71	^##^
**Q8R464**	CADM4	Cell adhesion molecule 4	1. Integrin signaling pathway	1.34	**	0.02	^##^
**Q4VAA2**	CDV3	Protein CDV3	——	0.36		-1.36	^#^
**P23953**	CES1C	Carboxylesterase 1C	1. Alzheimer disease-presenilin pathway	-1.70	**	-0.17	^##^
**Q9QUN9**	DKK3	Dickkopf-related protein 3	1. Wnt signaling pathway 2. p53 pathway	1.81	*	-0.52	^##^
**E9Q557**	DSP	Desmoplakin	1. Apoptosis signaling pathway 2. Interleukin signaling pathway	-0.18		2.09	^##^
**P62631**	EEF1A2	Elongation factor 1-alpha 2	1. PI3 kinase pathway	-0.34		1.95	^##^
**Q99M71**	EPDR1	Mammalian ependymin-related protein 1	1. Gonadotropin releasing hormone receptor pathway 2. Integrin signaling pathway	0.65	**	-0.20	^##^
**Q8R0W0**	EPPK1	Epiplakin	1. Cytoskeletal regulation by Rho GTPase	-0.05		2.61	^##^
**P49945**	FTL2	Ferritin light chain 2	1. Integrin signaling pathway	1.29	**	0.35	^##^
**Q02596**	GLYCAM1	Glycosylation-dependent cell adhesion molecule 1	1. Inflammation mediated by chemokine and cytokine signaling pathway	0.51	**	-0.31	^##^
**P24472**	GSTA4	Glutathione S-transferase A4	——	0.64		-1.19	^##^
**P62806**	HIST1H4A	Histone H4	——-	-0.41		2.32	^##^
**Q61696**	HSPA1A	Heat shock 70 kDa protein 1A	1. Apoptosis signaling pathway 2. Gonadotropin releasing hormone receptor pathway 3. Parkinson disease	-0.77	**	1.75	^##^
**P17879**	HSPA1B	Heat shock 70 kDa protein 1B	1. Apoptosis signaling pathway 2. Gonadotropin releasing hormone receptor pathway 3. Parkinson disease	-0.77	**	1.75	^##^
**Q02257**	JUP	Junction plakoglobin	1. Alzheimer disease-presenilin pathway	-0.54		2.14	^##^
**P28665**	MUG1	Murinoglobulin-1	——-	-1.12	**	0.05	^##^
**P02762**	MUP6	Major urinary protein 6	——-	1.67	*	0.77	
**P04938**	MUP8	Major urinary proteins 8	——-	1.67	*	0.77	
**Q5SX40**	MYH1	Myosin-1	1. Cytoskeletal regulation by Rho GTPase 2. Inflammation mediated by chemokine and cytokine signaling pathway 3. Nicotinic acetylcholine receptor signaling pathway 4. Wnt signaling pathway	1.49	**	1.27	^##^
**Q5SX39**	MYH4	Myosin-4	1. Cytoskeletal regulation by Rho GTPase 2. Inflammation mediated by chemokine and cytokine signaling pathway 3. Nicotinic acetylcholine receptor signaling pathway 4. Wnt signaling pathway	1.63		2.61	
**P13542**	MYH8	Myosin-8	1. Cytoskeletal regulation by Rho GTPase 2. Inflammation mediated by chemokine and cytokine signaling pathway 3. Nicotinic acetylcholine receptor signaling pathway 4. Wnt signaling pathway	1.44	**	1.41	
**Q8VDD5**	MYH9	Myosin-9	1. Cytoskeletal regulation by Rho GTPase 2. Inflammation mediated by chemokine and cytokine signaling pathway 3. Nicotinic acetylcholine receptor signaling pathway	1.09	*	3.44	^##^
**P05977**	MYL1	Myosin light chain 1/3, skeletal muscle isoform	1. Integrin signaling pathway	2.79	**	1.37	^#^
**Q99J85**	NPTXR	Neuronal pentraxin receptor	1. Alzheimer disease-presenilin pathway	-0.37		0.18	^##^
**P63054**	PCP4	Purkinje cell protein 4	1. Gonadotropin releasing hormone receptor pathway	4.32	**	2.26	^#^
**Q7TMJ8**	PIK3IP1	Phosphoinositide-3-kinase-interacting protein 1	1. PI3 kinase pathway 2. p53 pathway	0.42		-0.50	^##^
**P97350**	PKP1	Plakophilin-1	1. Cytoskeletal regulation by Rho GTPase 2. Apoptosis signaling pathway	-0.47		3.20	^##^
**Q9QXS1**	PLEC	Plectin	1. Cytoskeletal regulation by Rho GTPase 2. Integrin signaling pathway	0.52		2.72	^##^
**Q8K4L4**	POF1B	Protein POF1B	1. Ubiquitin proteasome pathway	-0.67		2.27	^##^
**Q9ERT9**	PPP1R1A	Protein phosphatase 1 regulatory subunit 1A	——	-0.30		-1.15	^##^
**Q61171**	PRDX2	Peroxiredoxin-2	1. p53 pathway 2. Apoptosis signaling pathway	1.01	*	-0.53	^##^
**O09114**	PTGDS	Prostaglandin-H2 D-isomerase	1. Alzheimer disease-presenilin pathway	-0.01		-0.58	^##^
**Q9WUB3**	PYGM	Glycogen phosphorylase, muscle form	1. Heterotrimeric G-protein signaling pathway-Gi alpha and Gs alpha mediated pathway	1.62		2.27	^##^
**P62983**	RPS27A	Ubiquitin-40S ribosomal protein S27a	1. Interleukin signaling pathway	1.35	**	-0.04	^##^
**P50114**	S100B	Protein S100-B	1. Inflammation mediated by chemokine and cytokine signaling pathway	0.01		-1.45	^##^
**P47867**	SCG3	Secretogranin-3	1. Alzheimer disease-presenilin pathway 2. Huntington disease	0.33		-0.49	^##^
**P70124**	SERPINB5	Serpin B5	1. p53 pathway	-1.07	*	3.44	^##^
**Q4V9Z5**	SEZ6L2	Seizure 6-like protein 2	1. Alzheimer disease-presenilin pathway	0.51	*	-0.44	^##^
**P54227**	STMN1	Stathmin 1	1. Cytoskeletal regulation by Rho GTPase 2. PI3 kinase pathway	1.69	**	-0.98	^##^
**P61957**	SUMO2	Small ubiquitin-related modifier 2	1. p53 pathway	0.73		-1.50	^##^
**Q9JLF6**	TGM1	Protein-glutamine gamma-glutamyltransferase K	——-	-0.59		1.19	^##^
**P68369**	TUBA1A	Tubulin alpha-1A chain	1. Gonadotropin releasing hormone receptor pathway 2. Cytoskeletal regulation by Rho GTPase	2.53	**	0.68	^##^
**P05213**	TUBA1B	Tubulin alpha-1B chain	1. Gonadotropin releasing hormone receptor pathway 2. Cytoskeletal regulation by Rho GTPase	2.53	**	0.68	^##^
**P68373**	TUBA1C	Tubulin alpha-1C chain	1. Gonadotropin releasing hormone receptor pathway 2. Cytoskeletal regulation by Rho GTPase	2.53	**	0.68	^##^
**P05214**	TUBA3A	Tubulin alpha-3 chain	1. Gonadotropin releasing hormone receptor pathway 2. Cytoskeletal regulation by Rho GTPase	2.53	**	0.68	^##^
**Q9D6F9**	TUBB4A	Tubulin beta-4A chain	1. Cytoskeletal regulation by Rho GTPase 2. Huntington disease	1.01		3.54	^##^
**P68372**	TUBB4B	Tubulin beta-4B chain	1. Cytoskeletal regulation by Rho GTPase 2. Huntington disease	1.19		3.48	^##^
**P0CG49**	UBB	Polyubiquitin-B	1. Huntington disease 2. Wnt signaling pathway 3. Ubiquitin proteasome pathway	1.35	**	-0.04	^##^
**P0CG50**	UBC	Polyubiquitin-C	1. Huntington disease 2. Wnt signaling pathway 3. Ubiquitin proteasome pathway	1.35	**	-0.04	^##^

*P < 0.05

**P < 0.01 compared to the control group.

#P < 0.05

##P < 0.01 compared to the CI/R group

### Bioinformatic analyses of BHD-responsive CSF proteins

The functional annotations of the 59 CI/R-changed proteins were analyzed using PANTHER (Protein Analysis Through Evolutionary Relationships)[17], by which proteins are classified according to the subcellular localization, molecular function, biological process, and related pathway. As expected, most (45.9%) proteins are located in the extracellular space, followed by the cytoplasm (37.9%) (S2A Fig). When categorized by molecular functions, over 90% of the CSF proteins are related to binding (28.4%), structural molecules (27.2%), catalytic activity (24.7%), and enzyme regulation (12.3%) (S2B Fig). These proteins were also categorized into different biological processes involving in the cellular process (29.3%), cellular component organization or biogenesis (18.9%), and metabolic process (18.9%) (S2C Fig). After that, we undertook a Gene Ontology (GO) enrichment analysis of 59 CI/R-changed proteins, processing genes in terms of their associated cellular component, molecular function and biological process. S3 Fig lists the top 6 significantly enriched GO terms (cellular component, molecular function and biological process) identified after screening with a threshold of false discovery rate (FDR) <0.05 and p value <0.05.

**Fig 2:**
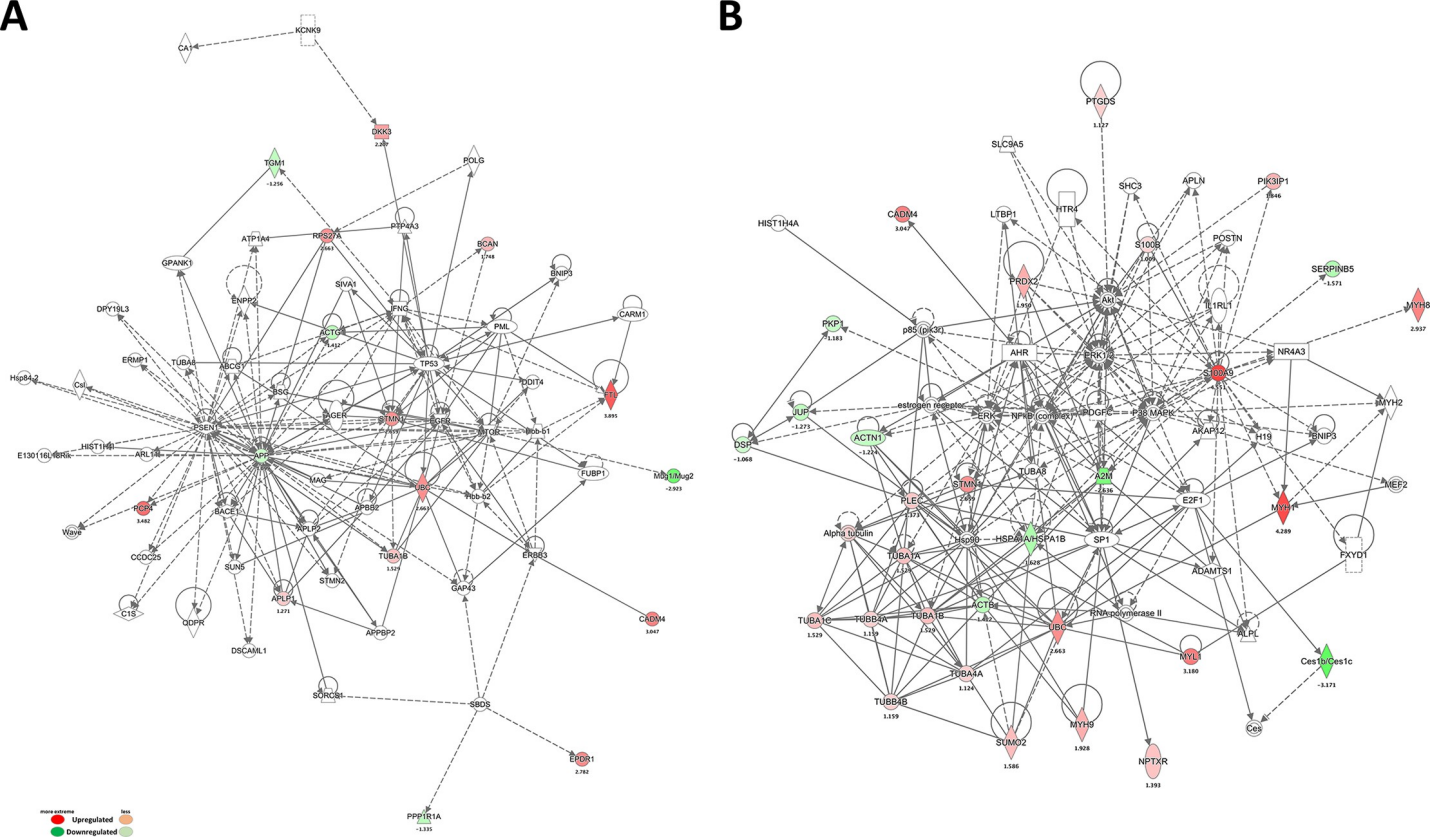
Protein-protein interaction network analysis of differentially expressed proteins. Network analysis was performed using ingenuity pathway tools (www.ingenuity.com) on proteins annotated in the Ingenuity database. (A) Neurological disease-related network and (B) inflammatory response-related network; the red nodes represent up-regulated proteins, and the green nodes represent down-regulated proteins.

Next, we attempted to identify the potential protein network(s) for the CI/R-changed CSF proteins using Ingenuity Pathway Analysis (IPA) (www.ingenuity.com). The candidate proteins were associated with two major protein networks (Fig 2). A number of direct or indirect associations between several “hub” proteins were placed at the center of this network. Of the hub proteins, A2M, APLP1, and STMN1 were identified in our study. Importantly, the proteins in these two protein networks are associated with neurological disorders (Fig 2A) and the inflammatory response (Fig 2B), indicating that BHD may regulate inflammation and neuroprotection.

To further assess this possibility, we also performed pathway analyses using PANTHER and IPA. According to prior studies, several pathways were correlated with pathology and repercussions of cerebral stroke [3, 26, 27], including cytoskeletal regulation by Rho GTPase (17.5%), inflammation mediated by the chemokine and cytokine signaling pathway (11.3%), Alzheimer disease-presenilin pathway (10.3%), and others (Table 1 and S2D Fig). Taken together, these proteomic data suggest mechanisms whereby BHD might be neuroprotective following stroke.

### Validation of BHD-responsive CSF proteins by Western blot

**Fig 3:**
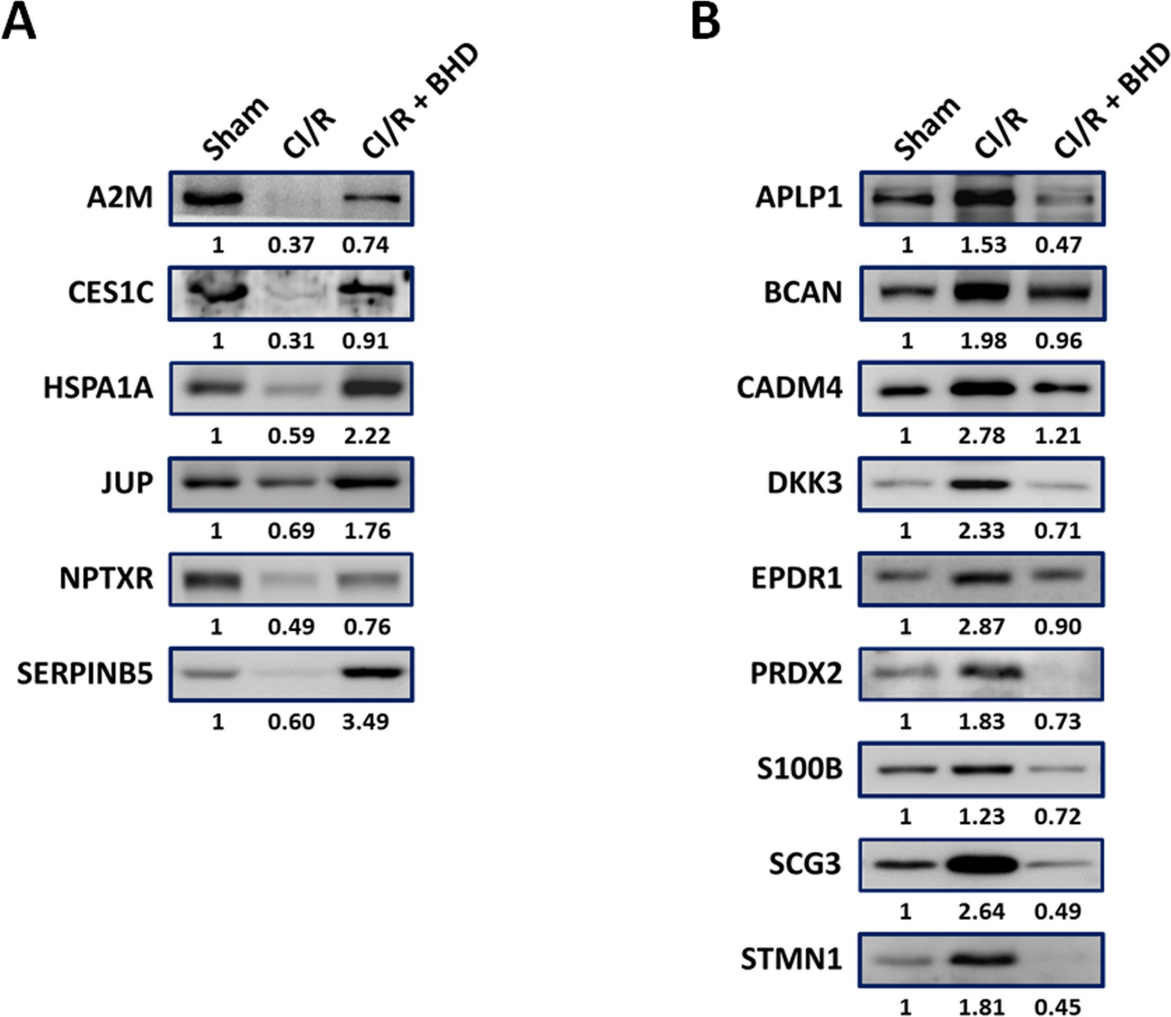
Validation of selected CSF proteins by Western blotting. (A-B) Mice were divided into three groups. One group served as untreated controls, one group had CI/R-induced stroke, and the other group was treated with CI/R and BHD. Mice were sacrificed and CSF was collected. The predicted markers were quantified followed by Western blot analysis.

Western blotting was used to validate the CI/R-changed protein candidates identified in the proteomics study. As shown in Fig 3A, several proteins were present in lower levels in the CI/R group, but were at near-normal levels in the C/IR+BHD group, including those involved in inflammation (A2M and SERPINB5) and neurodegenerative disorders (CES1C, HSPA1A, JUP, and NPTXR).

In contrast, many CSF proteins were up-regulated in CI/R mice (Fig 3B). APLP1 was one of these, which was consistent with our previous brain tissue study [9]. Two of these proteins, STMN1 and DKK3, were also found to be present at high levels in the CSF of AD patients [28, 29]. In addition, peroxiredoxin (PRDX) and S100B are well-known biomarkers in the CSF of acute stroke patients [30]. Furthermore, BHD treatment of CI/R mice was associated with candidate protein levels that tended to those of control mice (Fig 3B). In conclusion, the results of western blotting and proteomic analyses were well correlated (Table 1 and Fig 3). Combined our proteomics and Western blot data, these CI/R-changed CSF proteins regard as BHD-responsive CSF proteins.

### BHD-responsive metabolites changes in CI/R-mice

In foregoing classification and enrichment analysis, we revealed that BHD-responsive CSF proteins influence many terms, including metabolic process. Cerebral stroke also not only results in impaired function in part of the brain but also disrupts metabolism and brings about long-lasting changes that can be captured as metabolic signatures. Untargeted metabolomic analysis was first conducted using ^1^H-NMR spectroscopy to identify candidate metabolites in CSF. Ten metabolites (acetate, acetone, alanine, creatine, glucose, *myo*-inositol, *N*-nitrosodimethylamine, pyruvate, and succinate) showed remarkable differences between the CI/R and the sham groups (*p*<0.05) (Table 2). Of these, the levels of acetate, alanine, creatine, pyruvate, and acetone, were resemble to the results of the Wang *et al*. [31]. Besides, acetate, acetone, alanine, glucose, myo-inositol, and pyruvate, were also shown to be present in the brain tissues in our previous metabolomic study [10]. Meanwhile, acetone, glucose, and alanine were present at higher, and pyruvate was present at lower levels in the CI/R group. As shown in Table 2 and S5 Fig, BHD treatment could reverse a part of these metabolites in the CI/R-induced stroke mice. To identify additional metabolites, an UHPLC-QTOF-MS metabolomic study was further performed. This study attempted to evaluate 390 user-defined metabolites, of which 181 were detected and analyzed. Of these, 28 metabolites, including 1-methyladenosine, 2′-deoxyguanosine 5′-monophosphate, adenosine, and so on (Table 2), showed differences between treatment groups (*p*<0.05).

**Table 2:** CSF metabolites in CI/R-induced, BHD treatment, and sham mice by LC-MS/MS and ^1^H-NMR.

Metabolites name	LC-QTOF-MS		^1^H-NMR	
	CIR / Sham	CIR+BHD/ Sham	CIR / Sham	CIR+BHD/Sham
1-Methyladenosine	0.77 *	1.01 ^#^	N.D	N.D
2'-Deoxyguanosine 5'-monophosphate	2.94 *	2.38	N.D	N.D
Adenosine	0.46 *	0.52	N.D	N.D
Acetate	N.D	N.D	1.63 *	1.16 ^#^
Acetone	N.D	N.D	4.48 **	1.71 ^##^
Alanine	N.D	N.D	23.10 **	4.15 ^##^
Adenosine monophosphate (AMP)	2.78 **	2.48	N.D	N.D
Acetylcarnitine	1.16 *	2.22 ^#^	N.D	N.D
Creatine	2.09 *	0.86 ^#^	N.D	N.D
Creatinine	1.33 *	1.05 ^#^	1.64 **	1.27 ^##^
Cytidine	2.92 *	1.36 ^#^	N.D	N.D
Deoxycytidine	0.41 **	0.35	N.D	N.D
Dihydrouracil	4.61 **	3.00 ^#^	N.D	N.D
Dopamine	1.32 *	1.07	N.D	N.D
Glucose	N.D	N.D	1.46 *	1.15 ^#^
Glycerol 3-phosphate	6.71 **	6.13	N.D	N.D
Hypoxanthine	6.86 **	1.56 ^##^	N.D	N.D
Inosine	4.79 **	2.88 ^#^	N.D	N.D
Inosine-5'-monophosphate disodium salt (IMP)	3.43 **	4.58 ^#^	N.D	N.D
Isoleucine	0.68 *	1.23 ^#^	N.D	N.D
Levulinic acid	0.45 *	1.03 ^#^	N.D	N.D
Malic acid	3.62 **	1.38 ^##^	N.D	N.D
Methionine	1.57 **	1.09 ^#^	N.D	N.D
Myo-inositol	N.D	N.D	3.19 **	1.77 ^#^
N-Nitrosodimethylamine	N.D	N.D	0.70 *	0.68
Phenylalanine	1.51 *	1.15 ^#^	N.D	N.D
Pipecolic acid	0.80 *	1.13 ^#^	N.D	N.D
Propionyl-L-carnitine	2.60 *	1.80 ^#^	N.D	N.D
Pyroglutamic acid	0.61 *	0.57	N.D	N.D
Pyrrolidone carboxylic acid	0.64 *	0.68	N.D	N.D
Pyruvate	N.D	N.D	0.74 *	1.13 ^#^
Succinate	N.D	N.D	8.03 **	2.40 ^#^
Taurine	0.67 *	2.40 ^##^	N.D	N.D
Tyrosine	1.27 *	0.97	N.D	N.D
Xanthine	4.59**	1.36 ^##^	N.D	N.D
Uridine	11.19 **	1.83 ^##^	N.D	N.D

*P < 0.05

**P < 0.01 compared to the Sham group.

#P < 0.05

##P < 0.01 compared to the CIR group.

N.D.: no detected

Multivariate statistical methods (PCA, PLS-DA, and OPLS-DA) and unpaired *t*-tests were then used to identify the metabolites that were present in significantly different quantities among the three mice groups. Exploratory PCA analyses were employed to detect intrinsic clustering, as well as possible outliers, and the PC1 vs. PC2 scores plot (S4A Fig) showed differences in the concentration of several metabolites between the sham and stroke (CI/R) groups. By then applying PLS-DA, a reasonably good separation was obtained on the PC1 *vs*. PC2 plots (S4B Fig), with the CI/R group cluster being further away from the sham group cluster. Moreover, the BHD-treated (CI/R+BHD) group is closer to the sham group, suggesting that BHD treatment ameliorates the effects of CI/R on CSF metabolite concentrations. Finally, OPLS-DA was performed to minimize the possible contribution of intergroup variability and further improve the separation between the two groups. The OPLS-DA plot (S4C Fig) shows clear separation of metabolite concentrations for the sham, CI/R, and CI/R+BHD groups. Then, we conducted an OPLS-DA loadings plot analysis (S4D Fig), which showed some metabolites that contribute to the separation among sham, CI/R, and CI/R+BHD groups and demonstrated several crucial metabolites that were far from the center of the coordinate to indicate that these metabolites played an important role in clustering. In summary, UHPLC-QTOF-MS metabolomic analyses revealed significant differences in CSF metabolite concentrations (Table 2). Of these metabolites, isoleucine, which is a branched chain amino acid, was present at a lower levels following ischemic stroke [32], but this difference was abolished by BHD treatment (S5 Fig).

### Metabolic Pathway analyses of BHD-responsive metabolite changes

**Fig 4:**
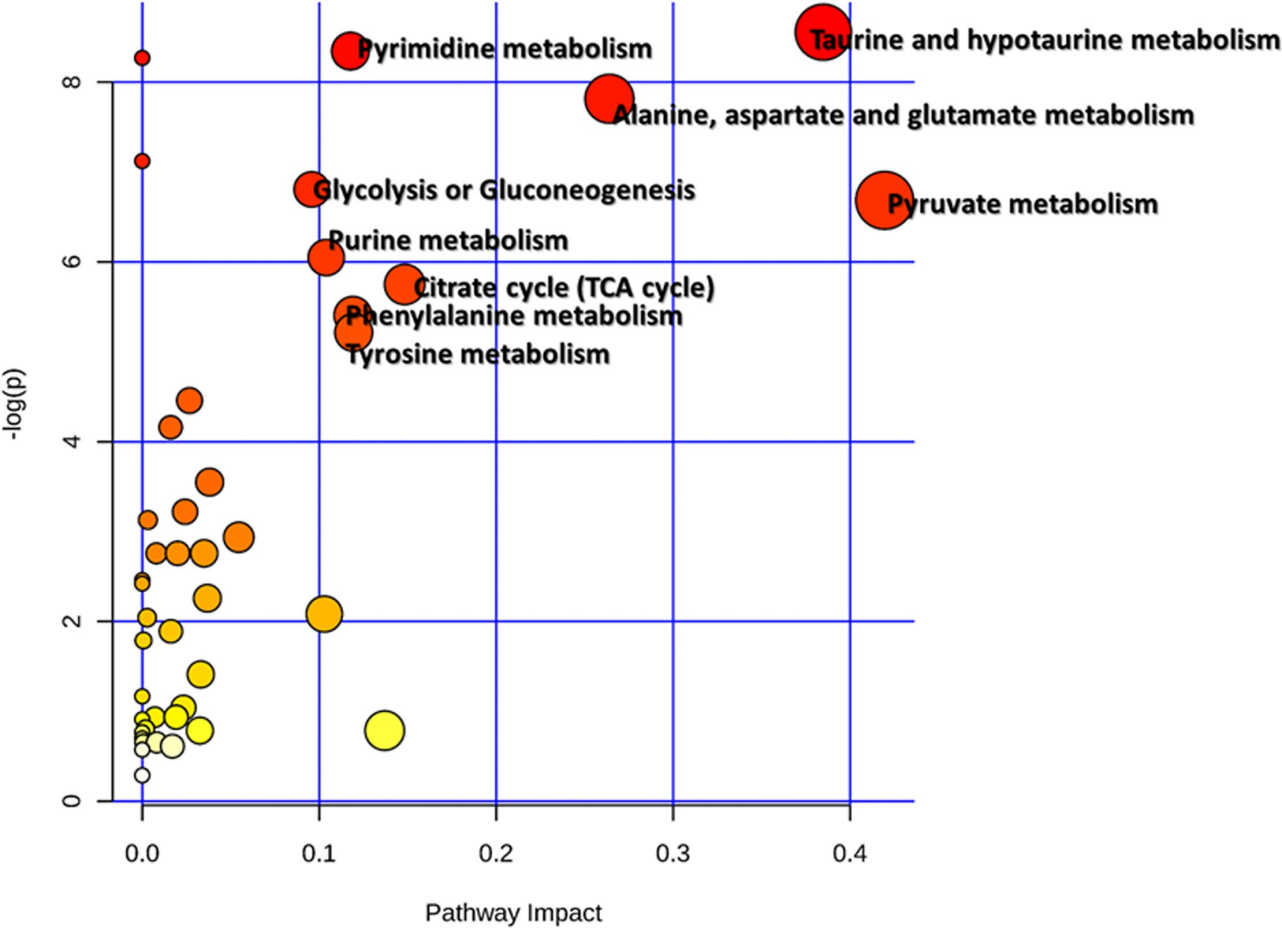
Metabolic pathways associated with BHD-responsive metabolites identifying network pathway by MetPA. The metabolism was inferred in the CI/R dosed mice from changes in the CSF levels of intermediates during substance metabolism.

To correlate the BHD-responsive metabolite changes, we next apply metabolomics pathway analysis (MetPA) [18] to identify the affected metabolic pathways. Nine disturbed metabolic pathways, including the taurine and hypotaurine metabolism, glycolysis or gluconeogenesis, citrate cycle (TCA cycle), alanine, aspartate and glutamate metabolism, pyruvate metabolism *etc*. were involved in CI/R-induced stroke and BHD treatment (impact > 0.1, *p* < 0.05) (Fig 4). These pathways are primarily related to the energy and amino acid metabolism.

## Discussion

Because of the intricacy and heterogeneity of stroke etiology, few biomarkers and therapeutic targets for ischemic stroke have been used in clinical practice. In this study, we have shown that the quantity of several proteins and metabolites in the CSF are affected by CI/R-induced stroke, and that these abnormalities are apparently ameliorated by BHD treatment. Most of these substances reflect inflammation and shifts in energy supply and demand, as well as pathophysiologic progress of neurodegenerative diseases.

Increasing evidence shows that metabolic changes in microenvironments are associated with diminished delivery and/or availability of oxygen, leading to inflammatory responses [33]. Extracellular purines and their degradation products, such as hypoxanthine and inosine, reach high concentrations in the extracellular space under inflammation stress and ischemia [34]. In addition, alterations in cellular energy homeostasis may contribute to ischemic and hypoxic brain damage. During cerebral ischemia, the brain preferentially utilizes creatine phosphate to generate adenosine triphosphate (ATP), leading to elevated CSF creatinine, which is a breakdown product of creatine phosphate, in stroke patients [35]. Hypoxanthine, xanthine, and uric acid levels in CSF have also been reported to be high in neurological patients, such as those with cerebral focal infarction or stroke, compared to healthy controls [36]. Our data are consistent with these previous findings, because the levels of inosine, creatinine, hypoxanthine, and xanthine were high in CSF samples from mice with CI/R-induced stroke.

On the other hand, acute inflammation also disrupts the blood-brain barrier (BBB) [37], leading to the release of cytoskeletal and cell adhesion proteins from dead neurons or other cells into CSF [38]. Our proteomics results show that many cytoskeletal proteins were present at higher-than-normal concentrations in CSF following stroke, including myosins (MYH1/4/8/9 and MYL1), and tubulins (TUBA1A/B/C, TUBA3A, and TUBB4A/B). In addition, we observed higher levels of ROS-related protein, glutathione *S*-transferase alpha 4 (GSTA4), which is a major end product of lipid peroxidation in stress-mediated signal transduction [39]. When the CSF metabolomic and proteomic data are considered together, we can infer that CI/R-induced brain damage involves loss of integrity of the BBB and acute inflammation, as well as alterations in energy homeostasis. CI/R injury followed by BHD treatment can reverse the levels of most inflammation-related proteins or metabolites in CSF. These results are consistent with our previous study on brain tissue [9].

Pluta and Zhang *et al*. found that transient CI/R-induced site-specific hyperphosphorylated Tau and increased brain immunoreactivity to the N- and C-terminal of amyloid beta precursor protein (APP) and Aβ in the ischemic cortex, implying that cerebral ischemia is involved in the pathogenesis of AD and contributes to the development of AD after stroke [40, 41]. Greater APLP1 and SCG3 expressions are associated with Aβ diffuse plaque and Aβ formation in AD patients [42, 43]. APLP1 expression levels are higher in vascular dementia (VaD) patients than in healthy controls [44]. BCAN, DKK3, HSPA1A, NPTXR, PRDX2, SERPINB5, STMN1, and other proteins in the CSF were also found alongside AD or VaD pathology [28, 45, 46]. Among these proteins, high expression of DKK3, which is a secreted protein in the prototypic Wnt/β-catenin/PKC pathway and P53 pathway, co-localizes with Aβ in senile plaques and strongly correlates with Aβ40 levels [47]. Our data also demonstrate that BHD treatment is associated with reductions in the high concentrations of DDK3 and the other neurodegeneration-related proteins listed above in CSF samples from CI/R mice. It is also worth noting that the concentration of APP, an important AD-related amyloid protein precursor [48], is decreased by BHD treatment. Further studies will be required to address the exact means whereby BHD regulates APP processing and protects against neurodegenerative diseases.

Our metabolomics analysis also revealed some amino acid-related metabolism pathways related to CI/R-induced stroke and neurodegenerative diseases. Czech *et al* and Martinez *et al* reported that amino acids including alanine, cysteine, dopamine, glycine, leucine, phenylalanine, proline, and tyrosine, were upregulated in CSF of patients with AD or VaD [49, 50]. These changes in amino acids may be the result of alternation in the Wnt/β-catenin signaling pathway [51], which was implied by our proteomic data. Further, high levels of acetate, creatinine, hypoxanthine, myo-inositol, and xanthine concentrations were measured in the CSF of dementia patients or the asymptomatic stages of AD [52–55]. Alterations in the taurine metabolism pathway and a lower levels of taurine were also found in the CSF of patients with neurodegenerative diseases, including AD, VaD, and PD [56, 57]. Interestingly, we have found evidence that BHD can overcome the reduction in taurine concentration, and even generate higher-than-normal taurine levels, in CI/R-induced stroke. In addition, other AD-related metabolites were also near-normalized by BHD treatment. Taken together, these data strongly suggest that BHD can ameliorate CI/R-induced stroke and may also protect against stroke-related AD.

In summary, we identified some protein candidates in the CSF of CI/R-induced stroke mice. Of these, APLP1, NPTXR, ACTN1 also were detected in the brain tissue of CI/R-induced stroke mice [9]. Likewise, acetate, acetone, alanine, glucose, *myo*-inositol, and pyruvate were identified in both the brain tissue and CSF of CI/R-induced-stroke mice. These common proteins and metabolites will be the focus of our further study. Taken together, the combination of proteomic and metabolomic data collected here have suggested mechanistic links between stroke and the pathogenesis of neurodegenerative diseases. These data may have diagnostic potential for stroke-related neurodegenerative diseases. Our data also imply that BHD treatment can reverse changes in inflammatory and neurodegenerative disease-associated biomolecules, and therefore potentially lower the risk of cerebral stroke-related neurodegenerative diseases. Some previous clinical reports have shown that BHD may be able to ameliorate neurodegenerative diseases, such as AD, VaD [58–60], but its mechanism is unclear. However, our data provide substantial new clues in this regard. In conclusion, BHD may be useful for the therapy of cerebral ischemia-associated diseases, and more importantly, it may have potential to lower the risk of neurodegenerative diseases. Further investigation is required to further clarify the mechanism whereby BHD may prevent neurodegenerative disease.

## Supporting information

S1 Table 344 proteins were quantitated from CSF. (XLSX)

S1 Fig The representative chemical fingerprint of BHD by UPLC. UPLC chromatogram was carried out on a Thermo Syncronis C18 column (2.1 mm×100 mm i.d., 1.7 μm) in Waters Acquity UPLC system with a diode array detector (DAD), monitor at 203, 230, and 280nm. The mobile phase was 0.1% phosphate water (A) and acetonitrile (B) with a program of 2% B at 0–1 min, 2–30% B at 1–10 min, 30–70% B at 10–15 min. The flow rate was 0.4 ml/min, and the column temperature was maintained at 35°C.
(TIF)

S2 Fig PANTHER analysis of proteins differentially expressed between CI/R-induced and control mice. (A) GO analysis of selected proteins in terms of cellular component (protein location). (B) PANTHER analysis of selected proteins in terms of molecular function. (C) PANTHER analysis of selected proteins in terms of biological process. (D) PANTHER analysis of selected proteins in terms of regulated pathways.
(TIF)

S3 Fig Functional enrichment analysis of the differentially expressed proteins. The y‑axis shows significantly enriched Gene Ontology (GO) terms relative to the genome, and the x-axis shows the fold enrichment of these terms. Red bars, “Molecular Function” categories in GO; green bars, “Cellular Component” categories in GO; gray bars, “Biological Process” categories in GO.
(TIF)

S4 Fig Metabolites changes among sham, CI/R and BHD groups. Scatter plots of scores of (A) PCA, (B) PLS-DA, and (C) OPLS-DA, obtained, respectively to the LC-QTOF-MS of CSF from sham (blue), CI/R (green), CI/R+BHD group (red). (D) Loading plot of OPLS-DA among sham, CI/R and BHD groups.
(TIF)

S5 Fig Quantitation of BHD-responsive metabolites in CSF by LC-QTOF-MS. (A) Metabolites with increased expression in the CI/R group and reversed with BHD treatment. (B) Metabolites with reduced expression in the CI/R group and reversed with BHD treatment. CIR: CI/R group; C+B: CI/R+BHD group. *P < 0.05, **P < 0.01 compared with the control group. #P < 0.05, ##P < 0.01 compared with the CI/R group.
(TIF)

S1 Text Details of method and materials and methods. (DOC)

## Abbreviations

HPLC: High performance liquid chromatography
NMR: Nuclear Magnetic Resonance
BHD: Buyang Huanwu decoction
TCM: traditional Chinese medicine
CSF: cerebrospinal fluid
CI/R: cerebral ischemic/reperfusion
VaD: Vascular dementia
AD: Alzheimer’s disease
PD: Parkinson’s disease
STAIR: Stroke Therapy Academic Industry Roundtable
IPA: Ingenuity Pathway Analysis
PANTHER: Protein Analysis Through Evolutionary Relationships
TPA: tissue plasminogen activator
MetPA: Metabolomics Pathway Analysis
SDS: sodium dodecylsulfate
PVDF: polyvinylidene difluoride
APLP1: amyloid beta precursor like protein
STMN1: stathmin 1
DKK3: dickkopf-related protein 3
CES1C: carboxylesterase 1C
SERPINB5: serpin family B member 5
A2M: alpha-2-macroglobulin
SCG3: secretogranin 3
BCAN: brevican
NPTXR: neuronal pentraxin receptor
CADM4: cell adhesion molecule 4
EPDR1: epiplakin 1
JUP: junction plakoglobin
S100B: S100 calcium binding protein B
PRDX: peroxiredoxin
ATP: adenosine triphosphate
GSTA4: glutathione *S*-transferase alpha 4
