# Supplementary figures and images for: Combined proteomic and metabolomic analyses of cerebrospinal fluid from mice with ischemic stroke reveals the effects of a Buyang Huanwu decoction in neurodegenerative disease
Source: PLoS One. 2019 Jan 15;14(1):e0209184. doi: 10.1371/journal.pone.0209184 (PMC6333407; doi:10.1371/journal.pone.0209184)

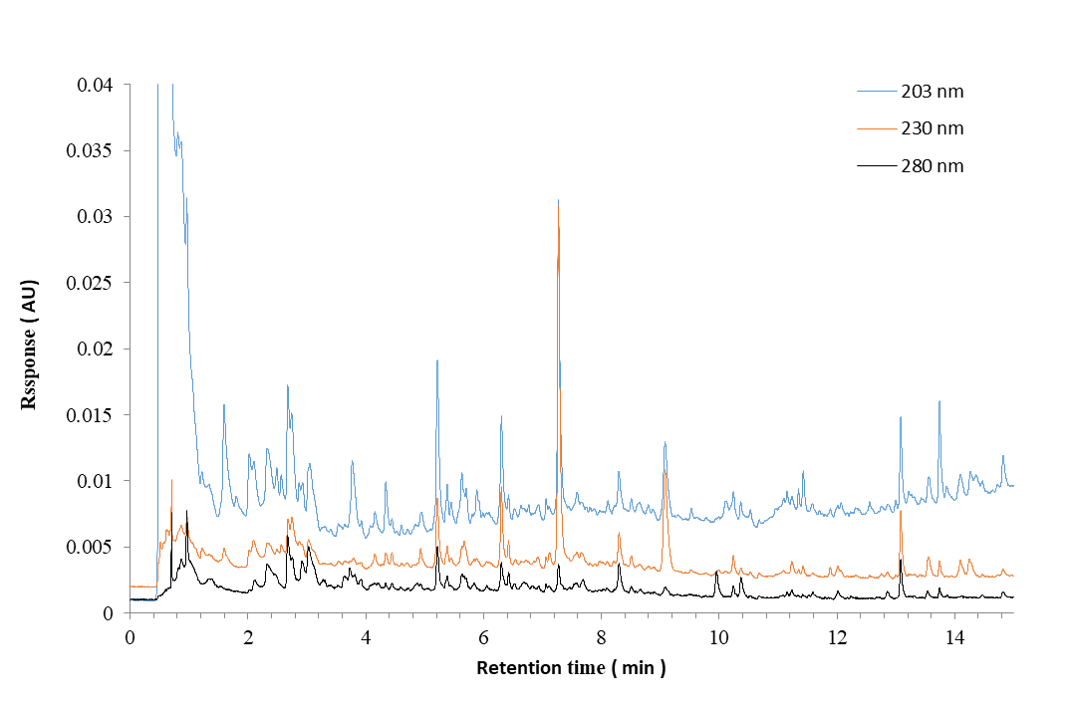

Supplement: S1 Fig — UPLC chromatogram was carried out on a Thermo Syncronis C18 column (2.1 mm×100 mm i.d., 1.7 μm) in Waters Acquity UPLC system with a diode array detector (DAD), monitor at 203, 230, and 280nm. The mobile phase was 0.1% phosphate water (A) and acetonitrile (B) with a program of 2% B at 0–1 min, 2–30% B at 1–10 min, 30–70% B at 10–15 min. The flow rate was 0.4 ml/min, and the column temperature was maintained at 35°C. (TIF) [file pone.0209184.s002.tif]

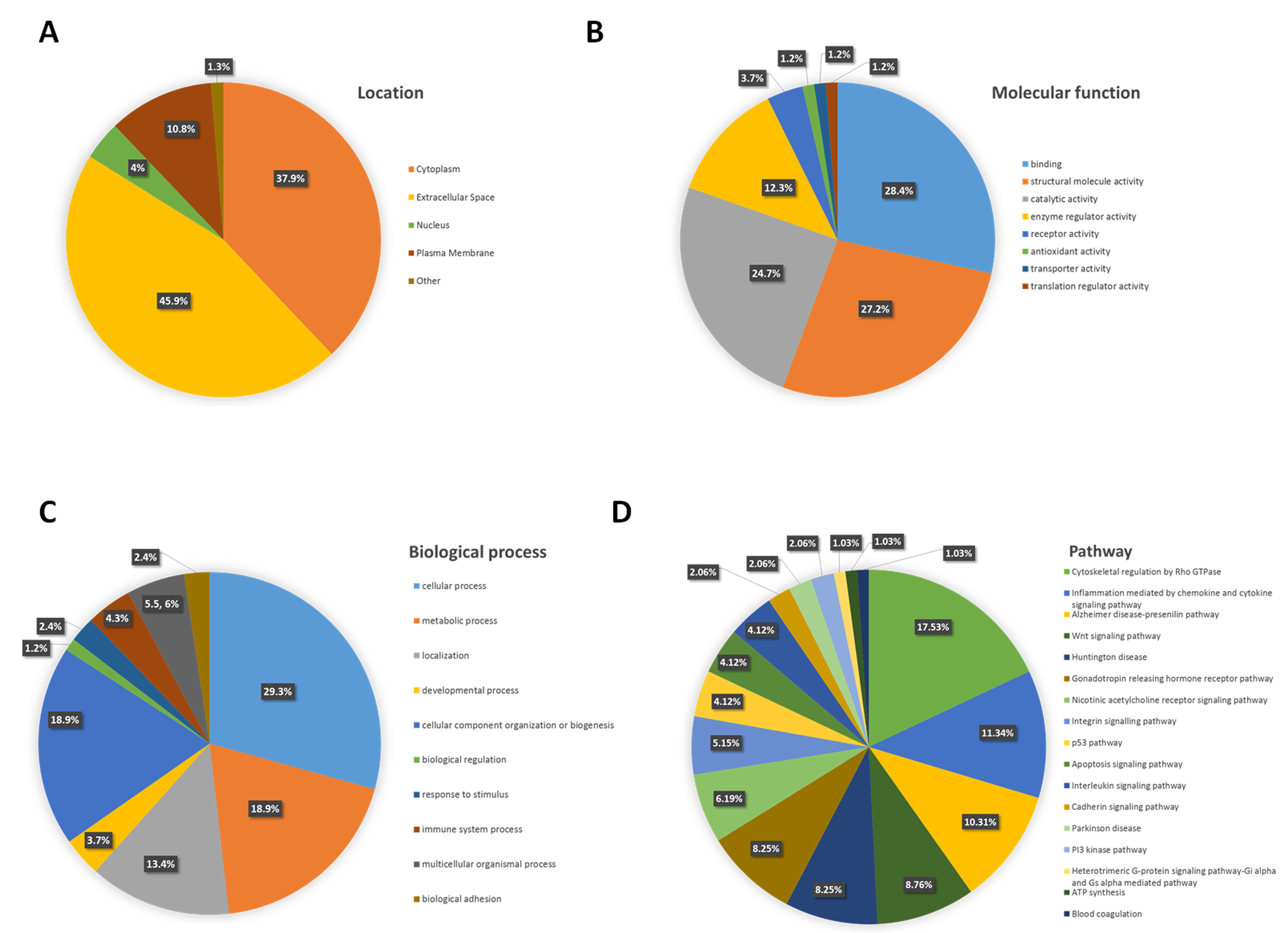

Supplement: S2 Fig — (A) GO analysis of selected proteins in terms of cellular component (protein location). (B) PANTHER analysis of selected proteins in terms of molecular function. (C) PANTHER analysis of selected proteins in terms of biological process. (D) PANTHER analysis of selected proteins in terms of regulated pathways. (TIF) [file pone.0209184.s003.tif]

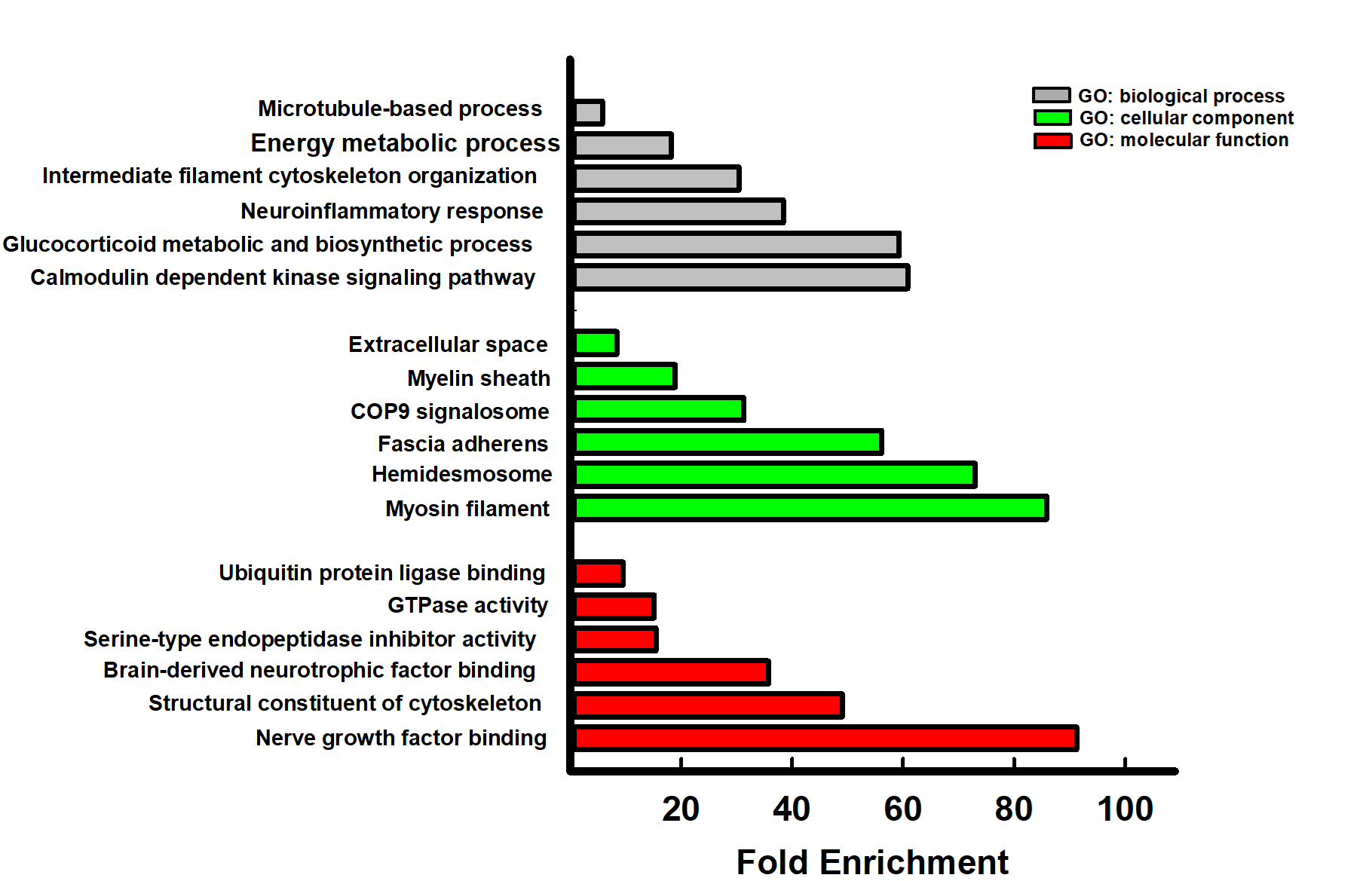

Supplement: S3 Fig — The y‑axis shows significantly enriched Gene Ontology (GO) terms relative to the genome, and the x-axis shows the fold enrichment of these terms. Red bars, “Molecular Function” categories in GO; green bars, “Cellular Component” categories in GO; gray bars, “Biological Process” categories in GO. (TIF) [file pone.0209184.s004.tif]

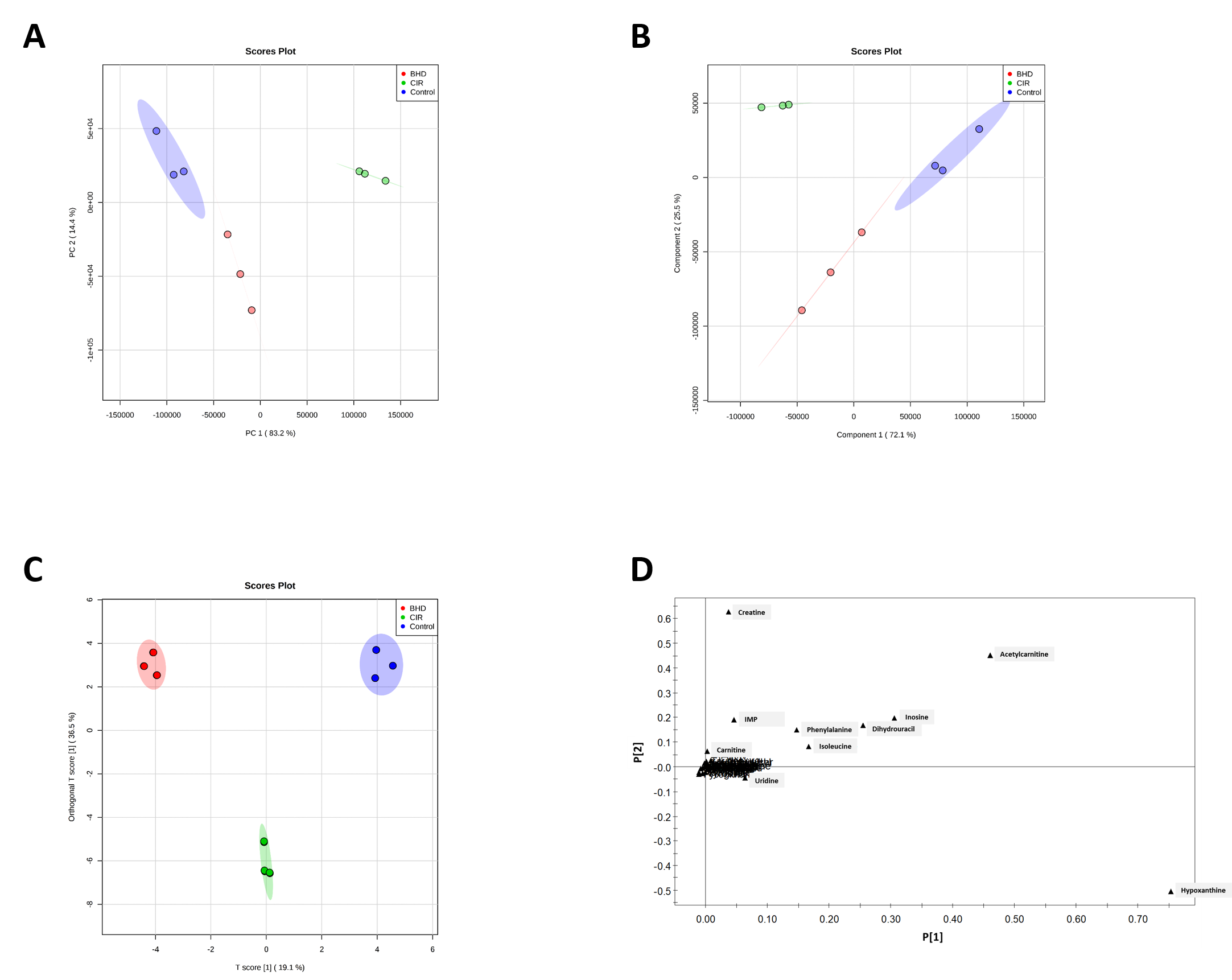

Supplement: S4 Fig — Scatter plots of scores of (A) PCA, (B) PLS-DA, and (C) OPLS-DA, obtained, respectively to the LC-QTOF-MS of CSF from sham (blue), CI/R (green), CI/R+BHD group (red). (D) Loading plot of OPLS-DA among sham, CI/R and BHD groups. (TIF) [file pone.0209184.s005.tif]

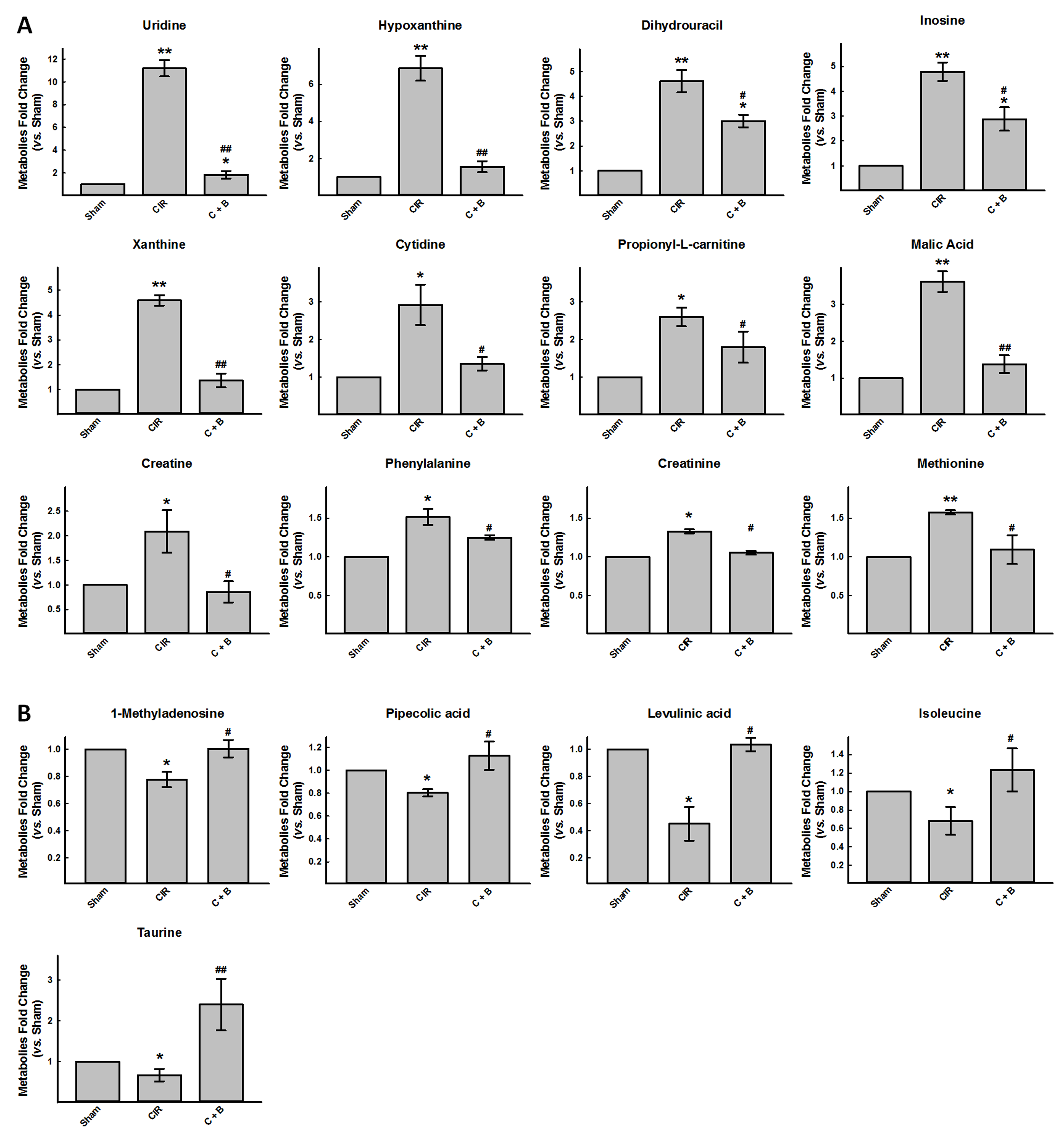

Supplement: S5 Fig — (A) Metabolites with increased expression in the CI/R group and reversed with BHD treatment. (B) Metabolites with reduced expression in the CI/R group and reversed with BHD treatment. CIR: CI/R group; C+B: CI/R+BHD group. *P < 0.05, **P < 0.01 compared with the control group. #P < 0.05, ##P < 0.01 compared with the CI/R group. (TIF) [file pone.0209184.s006.tif]
