# Supplementary material for: Combined proteomic and metabolomic analyses of cerebrospinal fluid from mice with ischemic stroke reveals the effects of a Buyang Huanwu decoction in neurodegenerative disease
Source: PLoS One. 2019 Jan 15;14(1):e0209184. doi: 10.1371/journal.pone.0209184 (PMC6333407; doi:10.1371/journal.pone.0209184)
Supplement: S1 Text — (DOC) [file pone.0209184.s007.doc]

**Supporting Information for details of method and Materials and Methods**

**Induction of cerebral ischemic/reperfusion (CI/R) injury**

The induction of middle cerebral ischemic/reperfusion (CI/R) injury, similar to the induction of middle cerebral artery occlusion (MCAO), was achieved using a previously reported method[1, 2]. The steps followed those recommended by the Stroke Therapy Academic Industry Roundtable (STAIR) [3]. Briefly, male mice (28-30 g) were anesthetized with a mixture of isoflurane (1.5% to 2%), oxygen, and nitrogen, and all efforts were made to minimize suffering. Humane endpoints were evaluated every 12 h after stroke induction. A fiber optic probe was glued to the parietal bone 2 mm posterior and 5 mm lateral to the bregma, and then connected to a laser-Doppler flowmeter (MBF3, Moor Instruments Ltd., Millwey, Axminster, UK) for continuous monitoring of the cerebral blood flow (CBF). For right middle cerebral artery (RMCA) occlusion in mice, a heat-blunted monofilament surgical suture (6-0) was inserted into the exposed external carotid artery, advanced into the internal carotid artery, and wedged into the Circle of Willis to obstruct the origin of the RMCA. The filament was left in place for 30 min and then withdrawn. Only animals exhibiting a reduction in CBF > 85% during RMCA occlusion and a CBF recovery > 80% after 10 min of reperfusion were included in this study. The average success rate of the surgery for the induction of ischemic stroke was around 80%. This procedure leads to reproducible infarcts that are similar in size and distribution to those reported in previous research using transient RMCA occlusion of comparable duration [1]. Rectal temperature was monitored and kept constant (37.0°C ± 0.5°C) during the surgical procedure and the recovery period, until the animal regained full consciousness.

**NanoLC-MS/MS analyses for proteomic study**

All nanoLC-MS/MS were performed on a Dionex Ultimate 3000 RSLC nano system (Thermo Fisher Scientific, Bremen, Germany) that was online coupled to an LTQ Orbitrap XL mass spectrometer (Thermo Fisher Scientific). Peptides were resuspended in 0.1% formic acid and loaded onto an in-house-prepared 100 μm × 15 cm tip column, which was packed with 3 μm ReproSil-Pur 120 C18-AQ reverse-phase beads (Dr. Maisch HPLC GmbH, Ammerbuch-Entringen, Germany). The LC mobile phases were 0.5% acetic acid in water (Buffer A), and a mixture of 0.5% acetic acid and 80% ACN (Buffer B). For peptide loading, the column was equilibrated at 5% Buffer B with a flow rate of 500 nL/min. The peptides were eluted at the flow rate of 500 nL/min with the following gradient: 5% to 10% Buffer B in 5 min, 10% to 40% Buffer B in 60 min, 40% to 99% Buffer B in 5 min, and 99% Buffer B in 10 min.

The LTQ Orbitrap XL system was operated under data-dependent acquisition (DDA) mode in the positive ion mode. Full-scan MS spectra (*m/z* 300–1600) were acquired on the Orbitrap analyzer with a resolution of 60,000 at *m/z* 400. For triggering DDA, the 10 most intense precursor ions with charge states ≥ +2 were automatically selected and fragmented using collision-induced dissociation in the linear ion trap with a normalized collision energy of 35%. The automated gain control (AGC) values for full MS and MS/MS were 5.0 × 10^5^ and 1.0 × 10^4^, respectively. A lock mass of *m/z* 445.120024 was applied in all LC-MS/MS analyses [4].

**Data analyses of CSF Proteomics**

Protein identification were done by MaxQuant software (version 1.5.2.8, Martinsried, Germany)[5] against the SWISS-PROT sequence database (version 2014_11 with 16,697 mouse sequence entries). Parent masses and fragment ions were searched with a mass tolerance of 4.5 ppm and 0.5 Da, respectively. The enzyme specificity was trypsin with up to two missed cleavages. Cysteine carbamidomethylation was set as a fixed modification, while N-acetylation of proteins and oxidation of methionine Variable modifications were set as variable modification. The minimum peptide length was set to 7 amino acids. To calculate the false discovery rate, all raw files were also searched against a decoyed, reverted mouse protein database, which was automatically generated by MaxQuant. The false discovery rate for both peptide and protein identifications were fixed at 1%.

Label-free quantitation (LFQ) information was calculated by the MaxLFQ algorithm [6], which was part of MaxQuant software. A minimum of 2 peptides were required for calculating the LFQ in each protein. In order to increase the quantitation information for each protein, the “match between run” function in MaxQuant was also used. The matching time window was 2 min and the alignment time window was 20 min. All raw files and MaxQuant-generated results were deposited to the ProteomeXchange Consortium [7] with the dataset identifier PXD006342.

The proteomic data were further analyzed by Perseus software (version 1.5.2.4, Martinsried, Germany) [8]. Before ANOVA analysis, each protein LFQ value was log2 transformed, and replicate were grouped. The statistical test was performed with a permutation-based false discovery rate (FDR) cutoff of 0.01 after 250 randomizations [8, 9].

**UPLC-QTOF-MS for metabolomic study**

UPLC-QTOF-MS for metabolomics were performed mainly according to the methods as previously described [10, 11]. Briefly, Agilent 1290-UHPLC coupled with Agilent 6540 Quadrupole-Time-of-Flight mass system (Agilent, Santa Clara, CA, USA) and ACQUITY UPLC HSS T3 column (100 Å, 2.1 × 100 mm, 1.8 μm, Waters) were employed. Mobile phase was solvent A (ACN/H2O/formic acid : 2/98/0.1, v/v/v), solvent B (ACN/formic acid: 1/1, v/v) with the program: 0-1 min 40% B; 1-5 min, 40-60% B; 5-6 min, 60-99% B; 6-7 min, 99% B; 7-8 min, 40% B; 8-9 min, 40% B. The injection volumn was 5 μl. The column temperature was 40℃, and the flow rate was 500 μL/min with the analytical time 9 min. For the sample preparation, CSF were vortexed with adequate MeOH/CAN/Acetone (1/1/1, v/v/v) for protein precipitation. After centrifugation (13000 *g*，4℃, 10 min), the supernatant was mixed with equal amount of acetone (at -20℃, 1 h), then centrifuged (13000 *g*，4℃, 10 min) again. The supernatant was lyophilized and stored at -80 ℃  until use.  For analysis, 100 μl MeOH/H_2_O (1/1, v/v) was added to the lyophilized sample and conducted analysis within 24 h. For sample ionization, a Jet Stream electrospray ionization source (Thermo Orbitrap Elite system (Thermo Fisher Scientific, Germaring, Germany)) was used with a capillary voltage of 4.0 kV in positive and negative modes. The MS parameters were set as follows: gas temperature, 325°C; gas flow, 5 L/min; nebulizer pressure, 40 psi; sheath gas temperature, 325°C; and sheath gas flow, 10 L/min. The MS scan range was set to *m/z* 50-1700. Each sample was performed duplicate.

**Figure Legends**

S1 Table 344 proteins were quantitated from CSF

S1 Fig. The representative chemical fingerprint of BHD by UPLC. UPLC chromatogram was carried out on a Thermo Syncronis C18 column (2.1 mm×100 mm i.d., 1.7 μm) in Waters Acquity UPLC system with a diode array detector (DAD), monitor at 203, 230, and 280nm. The mobile phase was 0.1% phosphate water (A) and acetonitrile (B) with a program of 2% B at 0-1 min, 2-30% B at 1-10 min, 30-70% B at 10-15 min. The flow rate was 0.4 ml/min, and the column temperature was maintained at 35°C.

S2 Fig. PANTHER analysis of proteins differentially expressed between CI/R-induced and control mice

(A) GO analysis of selected proteins in terms of cellular component (protein location). (B) PANTHER analysis of selected proteins in terms of molecular function. (C) PANTHER analysis of selected proteins in terms of biological process. (D) PANTHER analysis of selected proteins in terms of regulated pathways.

S3 Fig. Functional enrichment analysis of the differentially expressed proteins.

The y‑axis shows significantly enriched Gene Ontology (GO) terms relative to the genome, and the x-axis shows the fold enrichment of these terms. Red bars, “Molecular Function” categories in GO; green bars, “Cellular Component” categories in GO; gray bars, “Biological Process” categories in GO.

S4 Fig. Metabolites changes among sham, CI/R and BHD groups

Scatter plots of scores of (A) PCA, (B) PLS-DA, and (C) OPLS-DA, obtained, respectively to the LC-QTOF-MS of CSF from sham (blue), CI/R (green), CI/R+BHD group (red). (D) Loading plot of OPLS-DA among sham, CI/R and BHD groups.

S5 Fig. Quantitation of BHD-responsive metabolites in CSF by LC-QTOF-MS

(A) Metabolites with increased expression in the CI/R group and reversed with BHD treatment. (B) Metabolites with reduced expression in the CI/R group and reversed with BHD treatment. CIR: CI/R group; C+B: CI/R+BHD group. *P < 0.05, **P < 0.01 compared with the control group. #P < 0.05, ##P < 0.01 compared with the CI/R group.

S1 Text. Details of method and Materials and Methods

**Reference**

1. Chen HJ, Shen YC, Shiao YJ, Liou KT, Hsu WH, Hsieh PH, et al. Multiplex Brain Proteomic Analysis Revealed the Molecular Therapeutic Effects of Buyang Huanwu Decoction on Cerebral Ischemic Stroke Mice. PloS one. 2015;10(10):e0140823. doi: 10.1371/journal.pone.0140823. PubMed PMID: 26492191; PubMed Central PMCID: PMC4619651.

2. Chen HJ, Shen YC, Lin CY, Tsai KC, Lu CK, Shen CC, et al. Metabolomics study of Buyang Huanwu Tang Decoction in ischemic stroke mice by 1H NMR. Metabolomics. 2012;8(5):974-84. doi: 10.1007/s11306-011-0394-0.

3. Stroke Therapy Academic Industry R. Recommendations for standards regarding preclinical neuroprotective and restorative drug development. Stroke; a journal of cerebral circulation. 1999;30(12):2752-8. PubMed PMID: 10583007.

4. Olsen JV, de Godoy LM, Li G, Macek B, Mortensen P, Pesch R, et al. Parts per million mass accuracy on an Orbitrap mass spectrometer via lock mass injection into a C-trap. Molecular & cellular proteomics : MCP. 2005;4(12):2010-21. doi: 10.1074/mcp.T500030-MCP200. PubMed PMID: 16249172.

5. Cox J, Mann M. MaxQuant enables high peptide identification rates, individualized p.p.b.-range mass accuracies and proteome-wide protein quantification. Nature biotechnology. 2008;26(12):1367-72. doi: 10.1038/nbt.1511. PubMed PMID: 19029910.

6. Cox J, Hein MY, Luber CA, Paron I, Nagaraj N, Mann M. Accurate proteome-wide label-free quantification by delayed normalization and maximal peptide ratio extraction, termed MaxLFQ. Molecular & cellular proteomics : MCP. 2014;13(9):2513-26. doi: 10.1074/mcp.M113.031591. PubMed PMID: 24942700; PubMed Central PMCID: PMC4159666.

7. Deutsch EW, Csordas A, Sun Z, Jarnuczak A, Perez-Riverol Y, Ternent T, et al. The ProteomeXchange consortium in 2017: supporting the cultural change in proteomics public data deposition. Nucleic acids research. 2017;45(D1):D1100-D6. doi: 10.1093/nar/gkw936. PubMed PMID: 27924013; PubMed Central PMCID: PMCPMC5210636.

8. Tyanova S, Temu T, Sinitcyn P, Carlson A, Hein MY, Geiger T, et al. The Perseus computational platform for comprehensive analysis of (prote)omics data. Nature methods. 2016;13(9):731-40. doi: 10.1038/nmeth.3901. PubMed PMID: 27348712.

9. Tyanova S, Cox J. Perseus: A Bioinformatics Platform for Integrative Analysis of Proteomics Data in Cancer Research. Methods Mol Biol. 2018;1711:133-48. doi: 10.1007/978-1-4939-7493-1_7. PubMed PMID: 29344888.

10. Bruce SJ, Tavazzi I, Parisod V, Rezzi S, Kochhar S, Guy PA. Investigation of human blood plasma sample preparation for performing metabolomics using ultrahigh performance liquid chromatography/mass spectrometry. Analytical chemistry. 2009;81(9):3285-96. doi: 10.1021/ac8024569. PubMed PMID: 19323527.

11. Zelena E, Dunn WB, Broadhurst D, Francis-McIntyre S, Carroll KM, Begley P, et al. Development of a robust and repeatable UPLC-MS method for the long-term metabolomic study of human serum. Analytical chemistry. 2009;81(4):1357-64. doi: 10.1021/ac8019366. PubMed PMID: 19170513.
